# Evidence for Transmission of Bluetongue Virus Serotype 26 through Direct Contact

**DOI:** 10.1371/journal.pone.0096049

**Published:** 2014-05-05

**Authors:** Carrie Batten, Karin Darpel, Mark Henstock, Petra Fay, Eva Veronesi, Simon Gubbins, Samantha Graves, Lorraine Frost, Christopher Oura

**Affiliations:** 1 Non Vesicular Reference Laboratory, The Pirbright Institute, Woking, Surrey, United Kingdom; 2 Entomology, The Pirbright Institute, Woking, Surrey, United Kingdom; 3 Centre for Integrative Biology, The Pirbright Institute, Woking, Surrey, United Kingdom; 4 Vaccinology, The Pirbright Institute, Woking, Surrey, United Kingdom; 5 School of Veterinary Medicine, Faculty of Health and Medical Sciences, University of Surrey, Guildford, United Kingdom; 6 School of Veterinary Medicine, University of the West Indies, St. Augustine, Trinidad and Tobago; The University of Texas Medical Branch, United States of America

## Abstract

The aim of this study was to assess the mechanisms of transmission of bluetongue virus serotype 26 (BTV-26) in goats. A previous study, which investigated the pathogenicity and infection kinetics of BTV-26 in goats, unexpectedly revealed that one control goat may have been infected through a direct contact transmission route. To investigate the transmission mechanisms of BTV-26 in more detail an experimental infection study was carried out in which three goats were infected with BTV-26, three goats were kept uninfected, but were housed in direct contact with the infected goats, and an additional four goats were kept in indirect contact separated from infected goats by metal gates. This barrier allowed the goats to have occasional face-to-face contact in the same airspace, but feeding, watering, sampling and environmental cleaning was carried out separately. The three experimentally infected goats did not show clinical signs of BTV, however high levels of viral RNA were detected and virus was isolated from their blood. At 21 dpi viral RNA was detected in, and virus was isolated from the blood of the three direct contact goats, which also seroconverted. The four indirect barrier contact goats remained uninfected throughout the duration of the experiment. In order to assess replication in a laboratory model species of *Culicoides* biting midge, more than 300 *Culicoides sonorensis* were fed a BTV-26 spiked blood meal and incubated for 7 days. The dissemination of BTV-26 in individual *C. sonorensis* was inferred from the quantity of virus RNA and indicated that none of the insects processed at day 7 possessed transmissible infections. This study shows that BTV-26 is easily transmitted through direct contact transmission between goats, and the strain does not seem to replicate in *C. sonorensis* midges using standard incubation conditions.

## Introduction

*Bluetongue virus* (BTV) is the type species of the genus *Orbivirus*, family *Reoviridae* [1]. The BTV genome consists of 10 segments encoding 7 structural proteins and 4 non-structural proteins [2]. BTV is usually transmitted by biting midges (*Culicoides* spp.) [3] and can infect all species of ruminant [4], [5]. Clinical signs of BTV infection are more severe in naïve populations and are usually confined to sheep (particularly the improved meat and wool breeds) and white-tailed deer [6], [7]. The strain of bluetongue virus serotype 8 (BTV-8) which recently spread across Europe, was found to be transmitted transplacentally, orally and mechanically [8], [9] and also caused clinical signs in cattle and goats [10].

In early 2010, a novel strain of BTV was detected in a sheep and goat flock in Kuwait [11]. This virus was characterised as bluetongue virus serotype 26 (BTV-26) [11]. Experimental studies in sheep and goats revealed that five out of six experimentally infected sheep showed mild clinical signs characteristic of bluetongue, including conjunctivitis, reddening of the mouth mucosal membranes, slight oedema of the face and nasal discharge. Viral RNA was detected in 5 of the 6 sheep by real- time RT-PCR, however the levels of viral RNA detected in the samples were lower and of shorter duration than seen with other field strains of BTV [12]. Interestingly when 5 goats were experimentally infected with BTV-26, clinical signs of BTV were not observed, however high levels of viral RNA were detected and virus was isolated from the blood. One in-contact uninfected control goat was included in the study and 21 days into the experiment viral RNA was detected and virus was isolated from its blood [13]. These results showed that BTV-26 replicates to high levels in goats, suggesting that goats may be the natural host for this virus. The fact that one in-contact control goat was infected provided preliminary evidence indicating that BTV-26 may be spread by direct contact transmission.

Interestingly, it has not been possible to isolate BTV-26 in a KC - *C. sonorensis* embryo cell line [14] which is routinely used to isolate other field strains of BTV [11]–[13]. This indicates that this BTV-26 strain may not be adapted to insect cells and therefore may not be spread through *Culicoides* vectors. Similarly, it has so far proved impossible to isolate a strain of bluetongue serotype 25 (BTV-25) that was first identified to be circulating in goats in Switzerland [15], [16].

In this study we describe experiments carried out to investigate whether BTV-26 is transmitted through a direct contact route in goats. We also use oral infection of *Culicoides sonorensis* to assess whether or not *Culicoides* biting midges are likely to play a significant role in the transmission of BTV-26.

## Material and Methods

### 2.1 Viruses

The viral inoculum used for the experimental infection study was isolated from the blood of a BTV-26 infected sheep that had been passaged twice onto Baby Hamster Kidney (BHK) cells and confirmed as BTV-26 by serotype-specific real time RT-PCR [12].

### 2.2 Animals and Experimental Design

A group of 10 adult goats were used in the study. The goats were held in an insect-secure isolation unit at The Pirbright Institute and were under daily observation by veterinarians for the duration of the study.

**Figure 1:**
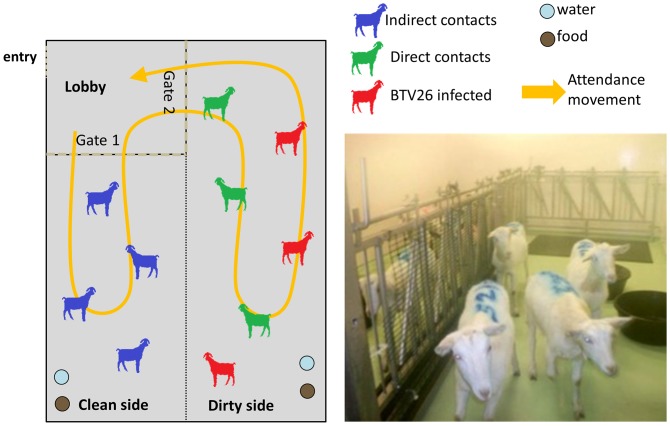
Animal facility layout and attendance management. The housing box was divided by a metal fence covered with a fine metal mesh. Both sides were accessible through a joint lobby and goats on each side had their own food and water facilities. The designated “dirty side” housed 3 goats which were subcutaneously inoculated with 1 ml of BTV-26 KUW2010/09 BHK_2_ as well as 3 uninfected ‘direct-contact’ goats. Four uninfected ‘barrier contact’ goats were housed on the clean side of the box. Animal attendants always entered and exited the clean side through the lobby before entering the dirty side and all sampling of animals was strictly carried out in the order ‘barrier goats’, ‘direct contact’ goats and finally infected goats. Furthermore the cleaning of the box was always carried out from the “clean side “to the “dirty side” towards the floor drains situated on the “dirty side”. Re-entering the clean side required disinfection and showering of waterproof personal protection equipment (PPE) or a change of PPE.

Three experimentally infected goats (animal numbers GT01–GT03) were inoculated subcutaneously with 1 ml of KUW2010/09 BTV-26 BHK2 at a titre of 10^6.0^TCID_50_/ml. These goats were housed in the same pen with 3 uninfected ‘direct contact’ goats (GT04–GT06) sharing food and water, as well as being exposed to pen mates' excreta (Figure 1 “dirty side”). The box was divided by a 1.50 m high metal fence which was additionally covered by fine metal mesh. Four uninfected ‘barrier contact’ goats (animal numbers GT07–GT10) were housed on the “clean” side of the pen (Figure 1- clean side). Close contact between the goats across this barrier was only possible either through sniffing on either side of the mesh or by standing on the hind legs to look over the barrier. The sharing of food, water and excreta between the dirty and clean sides of the pen was kept to a minimum by implementing a strict attendance and cleaning regime. Animal attendants would enter the clean side of the pen first via a lobby area (Figure 1) and the barrier contact goats were always attended to and sampled first. Additionally, cleaning was strictly carried out from the clean side to the dirty side and from the dirty side to floor drains in front of the lobby on the dirty side (Figure 1). Attendants would leave the clean side via the lobby and then enter the “dirty” side (Figure 1). The direct contact goats were always sampled before the experimentally infected goats. The body temperatures of each animal were recorded daily up to 14 days post infection (dpi) and the goats were examined daily for clinical signs. Clinical scoring was carried out using a Clinical Reaction Index modified from Huismans *et al* [17].

Blood samples (EDTA and whole blood) were taken at 0, 2, 4, 7, 9, 11, 14, 16, 18, 21, 23, 25, 28, 30 and 32 dpi. All animals were euthanized at the end of the experiment (32 dpi).

Non-invasive superficial swabs of secretions on the skin areas around the eye (ocular swabs) and nose (nasal swabs) were taken throughout the experiment.

#### 2.2.1 Ethical Statement

Experimental infections of the goats with bluetongue virus were approved by the Pirbright Institute ethics committee. All procedures were conducted in accordance with the UK Animals (Scientific Procedures) Act 1986, under project license permit numbers PPL 70/6798. To ameliorate suffering animals were observed at frequent, regular intervals and clinical score sheets were completed. Throughout the experiments the goats showed no clinical signs of disease.

### 2.3 Molecular analyses

#### 2.3.1 RNA extraction

RNA was extracted from 50 µl of EDTA blood, swab material supernatant (swab soaked in 500 µl PBS) and cell culture supernatant with a Universal (Qiagen, Crawley, UK) extraction robot using the ‘One for all’ protocol.

#### 2.3.2 Real time RT-PCR and quantification

Samples were analysed by BTV serogroup real time RT-PCR [18]. Copy number was determined using a molecular standard consisting of a dsDNA Ultramer Oligonucleotide (Integrated DNA Technologies, Inc, IDT, USA) designed to contain the probe sequence as described in Hofmann *et al.* 2008. Given that the exact molecular weight of the standard was known, copy number could be calculated.

### 2.4 Serological analyses

#### 2.4.1 ELISA

Whole blood samples were centrifuged at 2400 g for 5–10 minutes. Approximately 1–2 ml of serum was then collected into sterile microfuge tubes and stored at +4°C. BTV antibodies were detected using the BTV early detection ELISA (ID Vet, France). The assay was performed and analysed following the manufacturer's instructions.

#### 2.4.2 Serum Neutralisation Test (SNT)

SNT was performed as described [12].

### 2.5 Virus Isolation

EDTA blood cells were washed 3× with PBS and sonicated as described in the World Health Organisation (OIE) manual [19]. BHK cells were inoculated with 200–500 µl of washed blood or swab supernatant and incubated overnight. The following day the inoculum was removed and replaced with fresh media (DMEM, 1% pen/strep, 1% L-Glutamine). Cells were incubated for 7 days and then harvested by by cell scraping and centrifugation (2400 g for 5–10 minutes); supernatant was tested by real time RT-PCR for the presence of BTV RNA as described.

### 2.6 Vector competence studies

Approximately 500–600, 2–3 day old adult *C. sonorensis* biting midges (of the PIRB-s-3 line; [20] were deprived of sugar for 24 hours before being allowed to feed on a defibrinated horse-blood (TCS Biosciences, UK)/BTV-26 suspension (1∶2) via the Hemotek system (Hemotek Ltd, UK), using a Parafilmmembrane (Cole-Parmer, UK). The virus used (BTV-26 KUW2010/09 BHK_2_) had a titre of 10^5.83^ TCID_50_/ml prior to combination with the horse blood. Ten membrane fed *C. sonorensis* were processed immediately (day 0 post infection) and homogenized as whole insects using the Qiagen Tissue Lyser as previously described [21]. The remaining insects were incubated for 7 days at 25±1°C with access to 10% sucrose. At the end of the incubation period, 328 adult *Culicoides* were individually homogenized as whole insects in plain Schneider's *Drosophila* medium [21]. BTV RNA was extracted from the samples as described above (section 2.3.1). RNA was also extracted from 5 non-infected *C. sonorensis* as negative control.

## Results

### 3.1 Clinical observations

All three experimentally infected goats (GT01–GT03) showed a transient temperature rise of above 40°C between 7 and 10 dpi but no further clinical signs were observed in any of the goats (GT01–GT10) throughout the experiment. The uninfected direct contact goats (GT05 and GT06) showed transient temperature rises of above 40°C at 22 dpi (GT06) and 25 dpi (GT05). GT04 did not exhibit a rise in body temperature. The barrier contact goats (GT07–GT10) remained clinically normal throughout the experiment.

### 3.2 Pathology

At the end of the experiment (32 dpi) all 10 goats were euthanized and examined for pathological lesions. Goats GT01–GT06 had enlarged mesenteric lymph nodes, but no other pathological lesions were observed. No pathological lesions were observed in the barrier contact control goats (GT07–GT10).

### 3.3 Molecular analyses

BTV RNA was first detected by real time RT-PCR [18] in two of the experimentally infected goats (GT02 and GT03) at 2 dpi (copy number/ml of blood 2.59–9.78×10^2^) and in the third (GT01) at 4 dpi (copy number/ml of blood 5.06×10^3^). The peak of viraemia was observed in GT02 at 9 dpi (copy number/ml of blood 1.73×10^8^) and in GT01 and GT03 at 11 dpi (copy number/ml of blood 1.51×10^7^–1.01×10^8^) (Table 1). No BTV RNA was detected in the uninfected goats (GT04–GT10) at this time.

**Table 1:** BTV Real time RT-PCR threshold cycle (Ct) values and corresponding copy number/ml in blood and swab material collected from 3 goats (GT01–GT03) experimentally infected with BTV-26 and 3 direct contact goats (GT04–GT06).

Sample	dpi	GT01		GT02		GT03		GT04		GT05		GT06	
		Ct	copy number/ml	Ct	copy number/ml	Ct	copy number/ml	Ct	copy number/ml	Ct	copy number/ml	Ct	copy number/ml
Blood	0	No Ct	−	No Ct	−	No Ct	−	No Ct	−	No Ct	−	No Ct	−
Blood	2	No Ct	−	45.3	2.59×10^2^	44.3	9.78×10^2^	No Ct	−	No Ct	−	No Ct	−
Blood	4	34.5*	5.06×10^3^	32.2*	1.32×10^5^	33.6*	5.31×10^4^	No Ct	−	No Ct	−	No Ct	−
Blood	7	25.3*	2.36×10^6^	23.4*	4.80×10^7^	25.2*	1.42×10^7^	No Ct	−	No Ct	−	No Ct	−
Ocular Swab	7	No Ct	-	37.9*	2.98×10^3^	No Ct	-						
Nasal Swab	7	No Ct	-	No Ct	-	39.4	1.10×10^3^						
Blood	9	23.1*	1.17×10^7^	21.4*	1.73×10^8^	22.6*	7.96×10^7^	No Ct	−	No Ct	−	No Ct	−
Ocular Swab	9	No Ct	-	No Ct	-	No Ct	-						
Nasal Swab	9	37.1	5.03×10^3^	36.7	6.82×10^3^	36.5	7.68×10^3^						
Blood	11	22.6*	1.51×10^7^	22.5*	8.38×10^7^	22.2*	1.01×10^8^	No Ct	−	No Ct	−	No Ct	−
Ocular Swab	11	No Ct	-	No Ct	-	No Ct	-						
Nasal Swab	11	38.6	1.88×10^3^	37.1	4.98×10^3^	33.9	4.40×10^4^						
Blood	14	22.8*	1.29×10^7^	24.3*	2.55×10^7^	22.9*	6.14×10^7^	No Ct	−	No Ct	−	No Ct	−
Ocular Swab	14	No Ct	-	No Ct	-	No Ct	-						
Nasal Swab	14	34.9	2.27×10^4^	37.0	5.64×10^3^	35.9	1.11×10^4^						
Blood	16	25.1*	2.65×10^6^	28.8*	1.31×10^6^	24.7*	2.07×10^7^	No Ct	−	No Ct	−	No Ct	−
Ocular Swab	16	No Ct	-	No Ct	-	No Ct	-						
Nasal Swab	16	No Ct	-	38.5	2.08×10^3^	37.6	3.60×10^3^						
Blood	18	26.1*	1.33×10^6^	29.5*	8.09×10^5^	25.5*	1.17×10^7^	No Ct	−	No Ct	−	No Ct	−
Ocular Swab	18	No Ct	-	No Ct	-	No Ct	-						
Nasal Swab	18	39.2	1.24×10^3^	No Ct	-	No Ct	-						
Blood	21	28.4*	2.88×10^5^	29.9*	6.03×10^5^	30.1*	5.38×10^5^	43.6	2.38×10^3^	26.1*	6.30×10^6^	28.7*	1.99×10^6^
Ocular Swab	21	37.0	5.42×10^3^	No Ct	-	No Ct	-						
Nasal Swab	21	No Ct	-	No Ct	-	38.5	1.98×10^3^						
Blood	23	29.4*	1.45×10^5^	30.1*	5.57×10^5^	29.4*	8.71×10^5^	28.3*	1.78×10^6^	23.8*	7.07×10^7^	23.0*	8.59×10^7^
Ocular Swab	23	No Ct	-	No Ct	-	No Ct	-						
Nasal Swab	23	36.7	6.53×10^3^	33.5	5.47×10^4^	37.1	5.04×10^3^						
Blood	25	30.9*	5.49×10^4^	30.3*	4.79×10^5^	30.4*	4.26×10^5^	24.3*	2.44×10^7^	25.0*	3.26×10^7^	22.6*	1.14×10^8^
Ocular Swab	25	No Ct	-	No Ct	-	No Ct	-	No Ct	-	No Ct	-	38.7	1.78×10^3^
Nasal Swab	25	38.2	2.51×10^3^	No Ct	-	No Ct	-	No Ct	-	34.7	2.59×10^4^	38.4	2.15×10^3^
Blood	28	29.5*	1.40×10^5^	28.2*	1.91×10^6^	30.1*	5.42×10^5^	22.1*	1.13×10^8^	22.8*	1.35×10^8^	28.0*	3.34×10^6^
Ocular Swab	28	No Ct	-	No Ct	-	No Ct	-	No Ct	-	No Ct	-	No Ct	-
Nasal Swab	28	No Ct	-	No Ct	-	38.1	2.69×10^3^	35.3	1.72×10^4^	36.5	7.63×10^3^	32.2	1.30×10^5^
Blood	30	30.1*	9.34×10^4^	29.2*	1.05×10^6^	30.7*	3.53×10^5^	22.1*	1.15×10^8^	24.3*	5.22×10^7^	28.8*	1.87×10^6^
Ocular Swab	30	No Ct	-	No Ct	-	No Ct	-	No Ct	-	No Ct	-	36.8	6.19×10^4^
Nasal Swab	30	No Ct	-	No Ct	-	No Ct	-	37.3	4.45×10^3^	39.3	1.21×10^3^	38.5	1.96×10^3^
Blood	32	30.1*	9.52×10^4^	29.9*	6.13×10^5^	31.0*	2.98×10^5^	22.2*	1.04×10^8^	24.5*	4.48×10^7^	28.5*	2.38×10^6^
Ocular Swab	32	No Ct	-	No Ct	-	No Ct	-	36.0	1.09×10^4^	No Ct	-	No Ct	-
Nasal Swab	32	No Ct	-	No Ct	-	No Ct	-	No Ct	-	No Ct	-	No Ct	-

*Virus isolated directly on BHK.

Nasal and ocular swabs were collected from GT01–GT03 from 7 to 32 dpi. Low levels of BTV RNA were detected in the nasal swabs from 7–28 dpi with Ct values ranging from 33.5–39.4, corresponding to 1.10×10^3^–4.47×10^4^ BTV RNA copies/ml. BTV RNA was sporadically detected in the ocular swabs (Table 1).

At 21 dpi the uninfected direct contact goats (GT04–GT06) became real time RT-PCR positive with RNA detectable in their blood with Ct values ranging from 26.1–43.6, corresponding to 2.38×10^3^–6.30×10^6^ RNA copies/ml. The PCR performs within a linear range up to a Ct value of approximately 35 (unpublished data). Ct values decreased to 22 corresponding to a peak of greater than 1×10^8^ RNA copies/ml (Table 1). Nasal swabs from two goats were positive for BTV RNA at 25 dpi (GT05 and GT06) and at 28 and 30 dpi for all three goats (Ct values 32.2–39.3 corresponding to 1.21×10^3^–1.30×10^5^ BTV RNA copies/ml). RNA detection in the ocular swabs was less consistent (Table 1).

At the end of the experiment (32 dpi), all six experimental and direct contact infected goats (GT01–GT06) had detectable RNA in their blood as measured by real time RT-PCR, with Ct values ranging from 22.2–31.0 (Table 1). The uninfected barrier contact goats (GT07–GT10) were negative for BTV RNA throughout the duration of the experiment.

### 3.4 Serological analyses

Throughout the study BTV antibodies were measured by an early detection ELISA (data not shown). All three of the experimentally infected goats were seropositive by 7 dpi and the three direct contact control goats were seropositive at 28 dpi (data not shown). None of the barrier contact goats seroconverted.

All three of the experimentally infected goats (GT01–GT03) had detectable neutralising antibodies, measured by SNT, with titres (log_10_) at 32 dpi between 1.48–1.78. The three direct contact control goats (GT04–GT06) had no detectable neutralising antibodies in SNT at 32 dpi, which was not entirely surprising as these goats had only just seroconverted (as measured by ELISA) at 28 dpi.

### 3.5 Virus Isolation

Virus was isolated on BHK cells from blood samples from the experimentally infected goats (GT01–GT03) from 4 dpi. Virus was isolated from blood samples from two of the direct contact infected goats (GT05 and GT06) at 21 dpi and from the third direct contact infected goat (GT04) at 23 dpi. Virus was isolated from blood samples taken from all six experimentally and direct contact infected goats (GT01–GT06) at the end of the experiment (32 dpi). Cell culture supernatants were tested by real time RT-PCR for the presence of BTV RNA with Ct values ranging from 12.0 to 28.4. No virus was isolated from the blood of the barrier contact goats (GT07–GT10).

Nasal and ocular swabs were collected from experimentally infected goats (GT01–GT03) from 7–32 dpi and from direct contact goats (GT04–GT06) from 25–32 dpi i.e. after infection had occurred. Virus was isolated from an ocular swab from GT02 at 7 dpi. Cell culture supernatant was tested by real time RT-PCR for the presence of BTV RNA with a Ct value of 30.1. Virus was not isolated from any of the other swab samples which was not surprising due to the low levels of viral RNA detected in the swabs (Table 1).

### 3.6 Vector Competence

**Figure 2:**
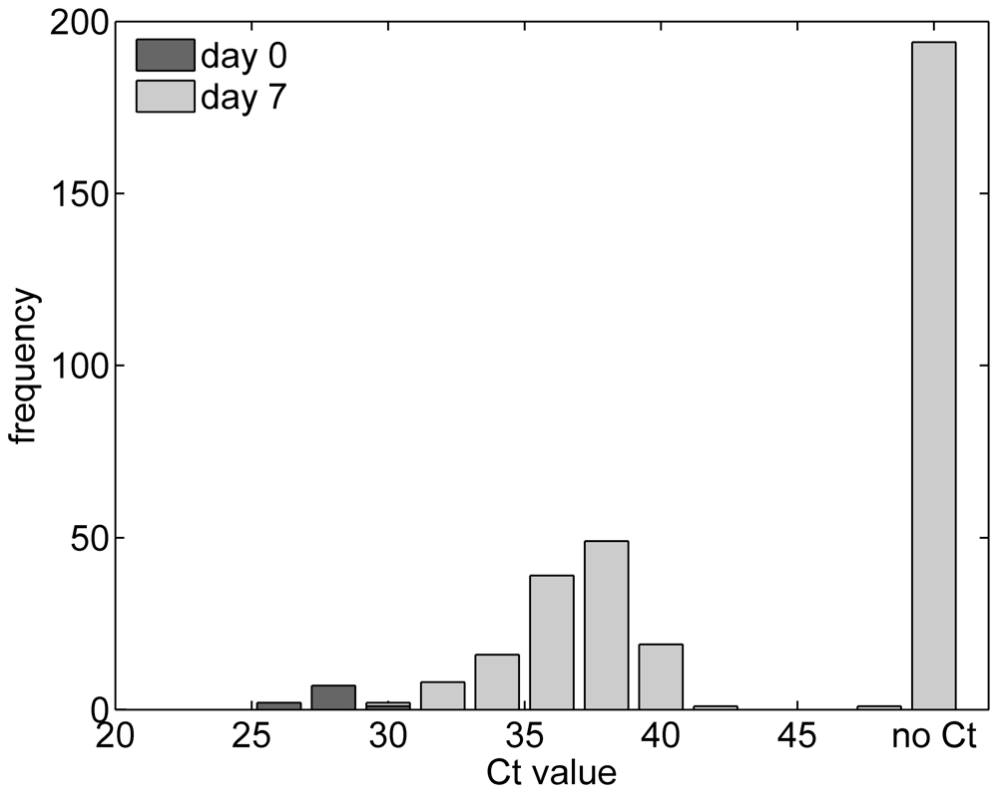
Observed Ct values for bluetongue virus (BTV) serotype 26 in *Culicoides sonorensis* fed on infected blood via a membrane. Individual midges were processed either immediately after feeding (day 0; dark grey bars) or after incubation at 25°C for seven days (day 7; light grey bars).

All ten *Culicoides* tested immediately after feeding (day 0) were positive for viral RNA (median Ct: 29.3; range 27.9 to 31.1 (Figure 2). Of the 328 insects incubated at 25°C for seven days, 134 had detectable levels of viral RNA (median Ct 38.11; range 31.8 to 48.7) (Figure 2), while the remaining 194 insects had no detectable viral RNA (i.e. no Ct value). The median Ct value of *Culicoides* tested on day 0 was significantly lower than the median for those tested after being incubated for seven days (Wilcoxon rank-sum test: *P*<0.001).

## Discussion

*Culicoides* biting midges are known to be the principal vectors responsible for BTV transmission in the field [4], [5], although there has been recent evidence showing that the European strain of BTV-8 was also capable of being transmitted through both the transplacental and the oral route [8], [9], [22], [23]. Two new serotypes of BTV (BTV-25 and BTV-26) have recently been identified [11], [24], which exhibit similar infection kinetics, with low levels of viral RNA detected for a short duration and mild clinical signs observed in sheep, and higher levels of viral RNA detected for a longer duration and no clinical signs observed in goats. [12], [13], [24]. Up to now there have been no reports of any BTV strains being transmitted through a direct contact route, however, the wide variations in seroprevalence for antibodies to BTV-25 observed in goat farms located in a similar region of Switzerland pointed to a possibility that BTV-25 was able to be transmitted by direct contact from goat to goat. No evidence of BTV-25 contact transmission was observed, however, when an uninfected control goat was housed with four experimentally infected viraemic goats [15]. Milk, urine, faeces and nasal and ocular swabs collected from the infected goats were negative by real time RT-PCR suggesting that BTV-25 was not excreted [15].

In a recent BTV-26 experimental infection study a single in-contact control goat was infected with BTV-26 at a time-point consistent with direct contact transmission of the virus. Low levels of viral RNA were detected in nasal swabs taken from two of the experimentally infected goats, indicating that these goats may have been excreting virus; however it was not possible to isolate virus from the PCR positive swab samples [13]. This result indicated that the in-contact control goat may have been infected through direct contact transmission. It was however impossible to draw definitive conclusions about direct contact transmission as only one in-contact control goat was included in the study. The aim of this current study was to carry out a more detailed investigation of BTV-26 transmission routes and to answer the two important questions: 1) Is BTV-26 transmitted through direct contact in goats? 2) Is a model species of *Culicoides* biting midge capable of becoming infected by BTV-26 and replicating the virus to transmissible levels using standard incubation conditions.

In order to address the first question of whether BTV-26 is likely to be transmitted through the direct contact route, three goats were experimentally infected with BTV-26, three goats were kept in direct contact and a further four goats were kept in indirect contact with the infected goats through a barrier. All three of the direct contact goats became infected at a time-point that was consistent with direct contact spread. Viral RNA was also detected in the nasal and ocular swabs taken from the experimental and direct contact infected goats. Although levels of viral RNA were low, it was possible to isolate virus from one ocular swab taken at 7 dpi from goat GT02. These results indicate that BTV-26 is easily transmitted through a direct contact route. The exact route of infection still needs to be confirmed, but the presence of viral RNA in both ocular and nasal swabs, and the positive virus isolation from one swab sample collected during peak infection points towards nasal or ocular secretions as a likely source of infection. Alternatively, oral infection of direct contact goats could have occurred through uptake of virus previously excreted into the shared water or food by the infected goats. Oral infection of BTV has already been observed in a mouse model, in carnivores and has been discussed as a possible route in ruminants [9], [25], [26]. Oral transmission of BTV or BTV RNA has been demonstrated in calves upon consumption of BTV positive colostrum [22], [23].

The four ‘barrier’ contact goats were not infected, indicating that close contact of the animals is necessary in order for the virus to be transmitted. Transmission is therefore likely to be through oral, nasal or ocular secretions, or possibly through the sharing of food and water, possibly by oral uptake of virus which would not have been possible for the barrier goats. Aerosol transmission of the virus, however, is unlikely. Other possible routes of infection are iatrogenic (through the use of shared needles), which can be ruled out in this experiment, and transmission through blood transfer from goat to goat, possibly through external wounds or fighting. The latter was considered extremely unlikely as the experimental goats were extremely placid and no fighting or wounds were observed on the goats throughout the study.

The second important question that has been addressed is whether BTV-26 is capable of replicating in a laboratory model *Culicoides* species. Previous studies in which *C. sonorensis* (of the same line as used in the present study) were infected via membrane feeding with either Schmallenberg virus (SBV) [27] or BTV [28]–[30] have shown that this species is competent to transmit both of these viruses. Following incubation for 7 days at 25°C all previously tested BTV strains have resulted in a proportion of *C. sonorensis* midges replicating the virus to transmissible levels [31]. The only notable exception has been the recently discovered BTV-25 strain which so far could not be isolated in vitro by any means tested, including oral feeding of *Culicoides* spp. [15]. Recent studies on the validation of the use of real time RT-PCR to infer vector competence for SBV or BTV have demonstrated that competence is indicated by a decrease in Ct value from a baseline on day 0 following incubation [27], [30]. Experimental infection of *C. sonorensis* with BTV-26 revealed that Ct values were significantly higher (indicating a reduced quantity of viral RNA) in midges tested following seven days' incubation, when compared with those tested immediately post feeding (Figure 2). This demonstrates that this BTV26 does not replicate in *C. sonorensis* in a comparable fashion to other BTV strains, either suggesting that this strain might require unusually long incubation periods or that *C. sonorensis* might not be a competent vector for this strain of BTV-26.

*C. sonorensis* is a Nearctic species and is not likely to be involved in transmission of BTV-26 in the field, however it remains the only primary BTV vector species that has been successfully colonised. Before it can be stated that *Culicoides* midges do not play a role in the transmission of BTV-26, competence studies should be carried out on field-collected *Culicoides* populations, using either Palaearctic species (to assess the risk of BTV-26 to European livestock) or the afrotropical vector *Culicoides imicola* (to assess competence of the predominant vector species where BTV-26 was first isolated in Kuwait). These studies, however, are significantly more challenging to perform [32], [33].

This is the first report of any BTV strain being transmitted through direct contact and is a highly significant and potentially worrying finding considering the known potential of co-circulating BTV strains to undergo reassortment in the field [34]. Reassortment between a co-circulating highly virulent BTV strain, such at the recent European strain of BTV-8, and a BTV strain capable to being transmitted by direct contact (BTV-26), could result in the generation of a virulent strain that is capable of being transmitted by both *Culicoides* midges as well as direct contact. More detailed work is required, for example using reverse genetics techniques [35], in order to understand the molecular mechanisms controlling the transmission of BTV and this new strain of BTV-26 will aid significantly in this endeavour.

Furthermore, several BTV strains have demonstrated successful transmission between mammalian hosts via alternative pathways (oral, transplacental) in addition to the transmission by *Culicoides* species. It is therefore important to investigate the potential alternative transmission mechanisms for any newly emerging BTV strains, because control strategies may be severely hampered if epidemiologically important alternative transmission pathways are not identified.
